# Pleural, pancreatic and prostatic involvement in IgG4-related disease mimicking pancreatic head malignancy

**DOI:** 10.1259/bjrcr.20190110

**Published:** 2020-09-29

**Authors:** Robert W Foley, Stewart L Redman, Richard N Graham, Jaspal S Phull, Vidan Masani, Benjamin J Colleypriest, David Little

**Affiliations:** 1 Department of Radiology, Royal United Hospital, Bath, United Kingdom; 2 Department of Urology, Royal United Hospital, Bath, United Kingdom; 3 Department of Respiratory Medicine, Royal United Hospital, Bath, United Kingdom; 4 Department of Gastroenterolgy, Royal United Hospital, Bath, United Kingdom

## Abstract

We describe the case of a gentleman with pleural thickening. On follow-up imaging, dilatation of the main pancreatic and common biliary ducts was noted and an initial diagnosis of pancreatic malignancy was made. During his preoperative workup for pancreatic head malignancy, a PET-CT was performed, which demonstrated increased uptake in the pancreas, in the pleura and in the prostate gland. This raised the possibility of immunoglobulin G4-related disease (IgG4-RD), which was effectively treated with oral steroids. IgG4-RD is a well-described cause of autoimmune pancreatitis but can affect other regions, including the pleura and prostate. It is essential that radiologists are aware of the imaging findings in IgG4-RD and can direct clinicians towards this important multisystem diagnosis.

## Clinical presentation

**Figure 1.**
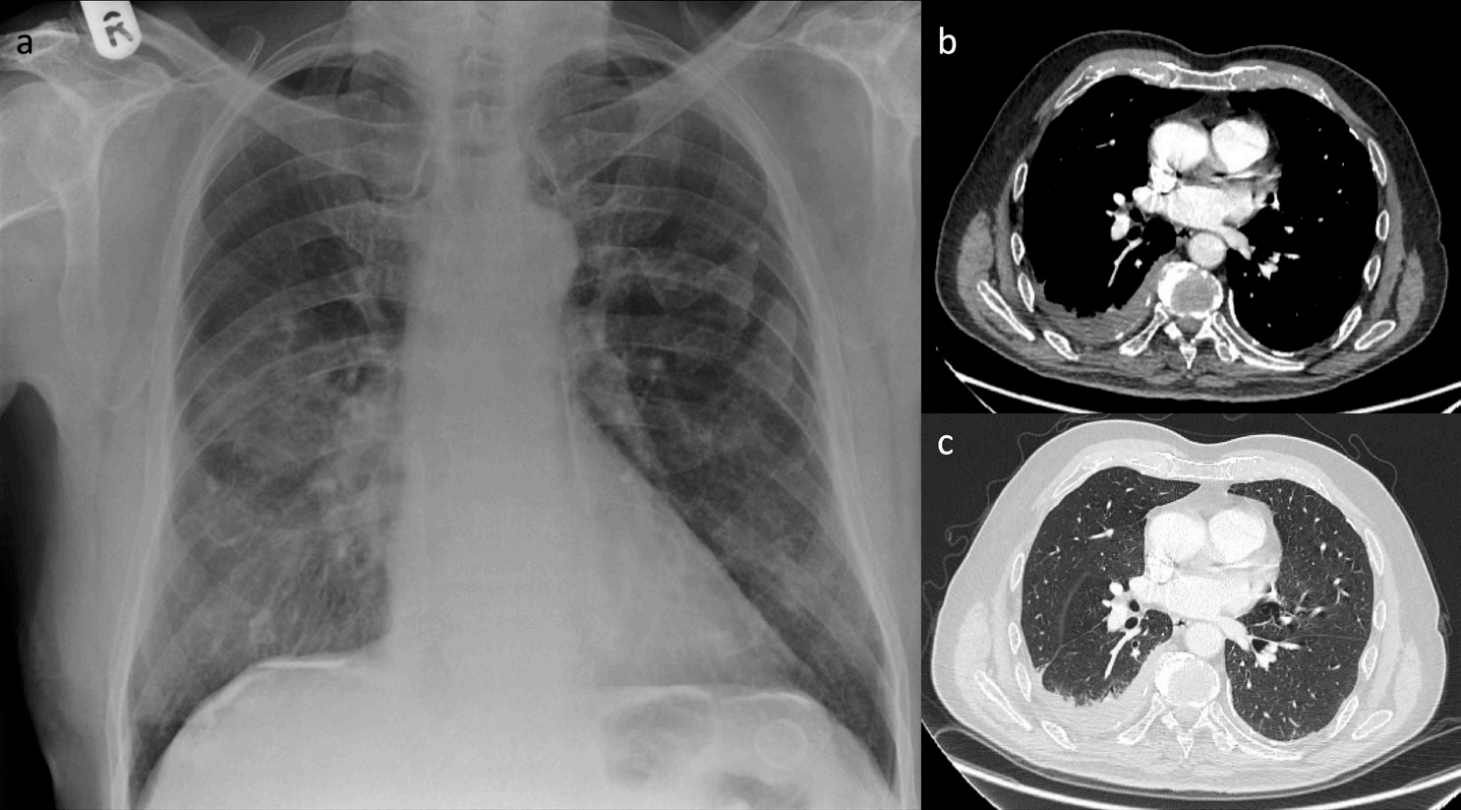
Initial chest radiograph (a) was remarkable for pleural plaques and increased density adjacent to the right hilum. A CT chest was subsequently performed with irregular right-sided pleural thickening illustrated on mediastinal (b) and lung windows (c)

A 76-year-old male presented to his general practitioner (GP) with a 4-week history of dry cough. He was a lifelong non-smoker, with a past medical history of non-muscle invasive bladder cancer and sigmoid cancer. He was a carpenter and had previously worked with asbestos sheets. His GP requested a chest X-ray, which demonstrated pleural calcification and a new rounded density in the right mid-zone that was not present on the previous imaging (Figure 1a). A CT scan of the thorax was advised, which demonstrated peripheral pulmonary consolidation and pleural thickening over the posterior right chest wall (Figure 1b and c).

**Figure 2.**
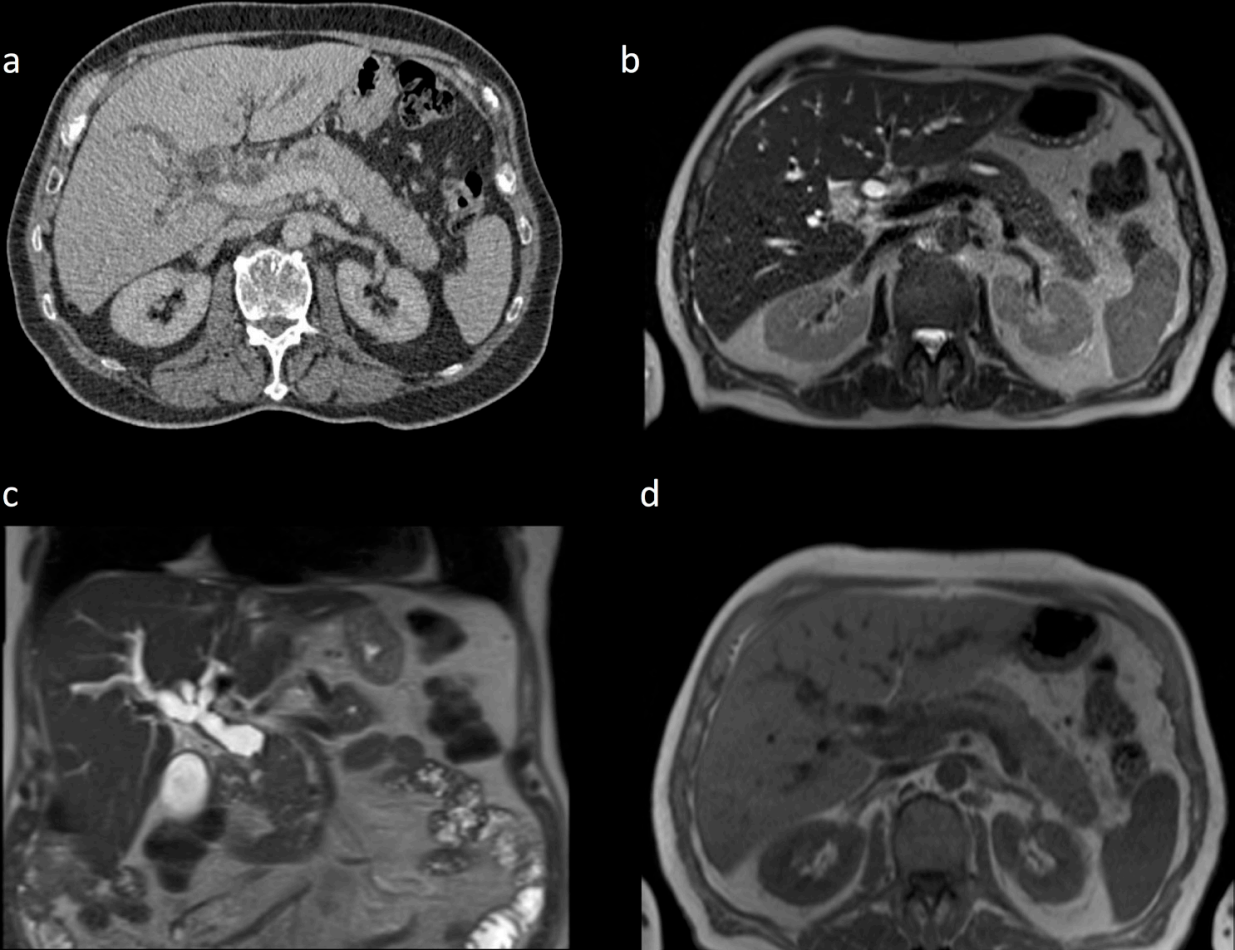
Dilatation of the main pancreatic duct on portal venous phase CT (a) and on T2W MRI (b). Dilatation of the common bile duct and more proximal biliary tree is illustrated on this coronal T2W image (c). T1W MRI demonstrates pancreatic oedema and a peripheral rim of low signal in the tail of the pancreas (d)

The patient was treated with antibiotics, seen in respiratory clinic and scheduled for CT surveillance of his pulmonary consolidation and pleural disease. Three-month, six-month and twelve-month follow-up CT scans demonstrated stable pleural disease, however prior to the 12-month scan the patient had developed deranged liver function tests and the abdomen was included on the subsequent CT. The CT, acquired in the portal venous phase, demonstrated dilatation of the proximal portion of the pancreatic duct within the head of pancreas, and a normal calibre duct more distally. There was associated intra-hepatic and extra-hepatic biliary dilatation, which were normal on previous imaging (Figure 2a). The pancreatic head demonstrated normal enhancement, iso-attenuating to the normal background pancreatic parenchyma, however of note, the CT did not include pancreatic arterial phase imaging. There was no invasion of the adjacent superior mesenteric vein, superior mesenteric artery or portal vein, which were all patent. These appearances were suspicious for a pancreatic head malignancy and so the patient was referred to upper gastrointestinal (UGI) clinic, where it was noted the patient’s appetite was poor, he was losing weight and his blood tests confirmed an obstructive jaundice.

Following discussion at the UGI multidisciplinary team meeting and an initial diagnosis of pancreatic malignancy, an MR pancreas and staging PET-CT were arranged to further investigate and evaluate disease extent. MR demonstrated pancreatic and biliary duct dilatation (Figure 2b–d), with a low signal focus in the pancreatic head on T1, which was isointense to normal pancreatic parenchyma on T2 and diffusion-weighted imaging.

**Figure 3.**
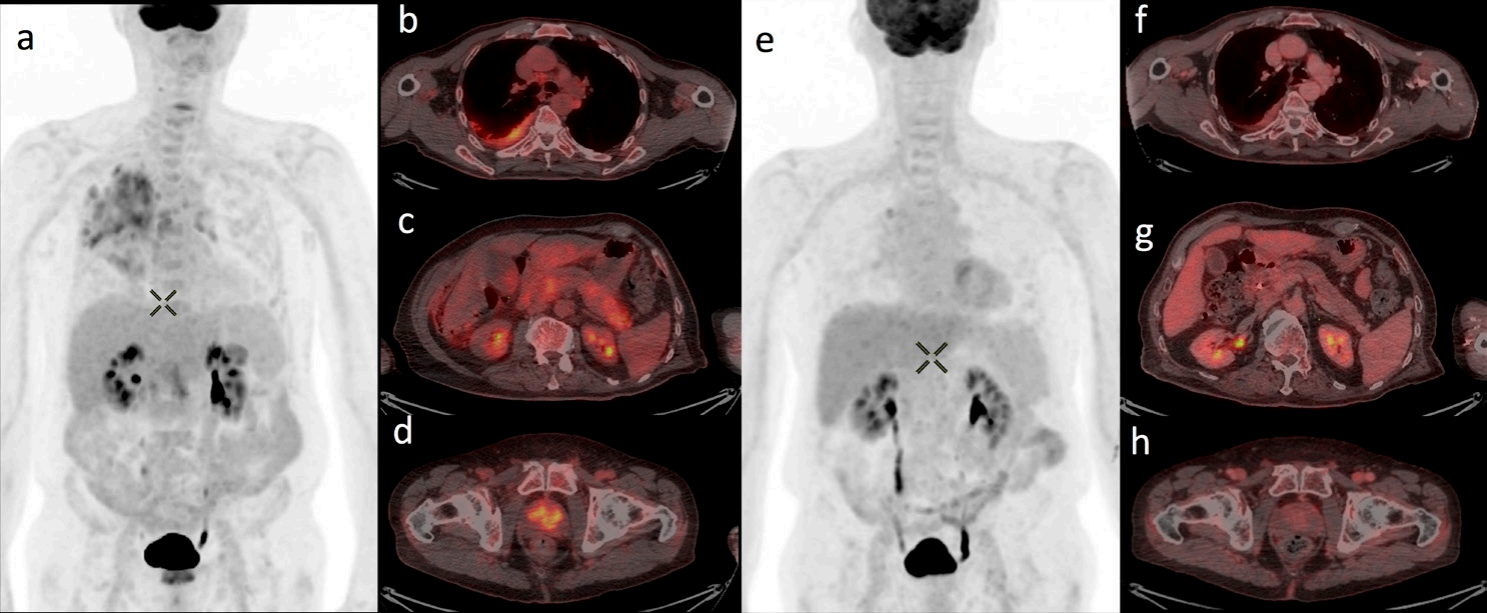
FDG PET-CT maximum intensity projection images before (a) and 10 weeks following initiation of steroid therapy (e). On the pre-treatment scan, there is avid FDG uptake within the right pleura (b), head and tail of the pancreas (c) and prostate gland (d). The post-treatment scan demonstrates decreased uptake in each of these areas (f, g, h)

Fluoro-deoxyglucose (FDG) PET-CT was performed (Figure 3), which demonstrated an enlarged pancreas with diffuse increased FDG uptake in the head (SUV Max 5.9) and tail (SUV Max 5.3), with sparing of the neck. There was also increased uptake within the right pleura (SUV Max 6.9) and diffuse increased uptake throughout the peripheral and central zones of the prostate.

## Differential diagnosis

The differential diagnosis for this patient’s pleural thickening was a pleural malignancy, especially in light of the patient’s previous asbestos exposure, while inflammatory causes and infection were the other main differentials. The patient’s pancreatic and biliary duct dilatation were suspicious for malignancy and the main differential was a pancreatic head or ampullary tumour; IgG4-related pancreatitis was considered a differential. The diffuse changes seen in the prostate gland were unusual for malignancy, in that the peripheral zone was spared, and so prostatitis was the main differential.

## Further investigations

Serum IgG4 levels were requested and endoscopic retrograde cholangio-pancreatography (ERCP) was performed. Endoscopic ultrasound demonstrated a mass in the pancreatic neck and body, and biopsies of the ampullary region were performed. A cholangiogram demonstrated a common bile duct stricture with intrahepatic regions of alternating strictures and dilatation. A stent was placed in the common bile duct and brushings were taken. The patient’s serum IgG4 was raised at 160 mg dl^−1^, while total IgG was also elevated at 221.8 mg dl^−1^. Histology demonstrated inflammatory changes only and the samples were IgG4-positive on immunohistochemistry.

## Treatment and outcome

Steroid treatment was commenced with oral prednisolone 30 mg once daily. A repeat PET-CT was undertaken 10 weeks following steroid therapy, illustrated in Figure 3. The patient had a good response with decreased tracer uptake in the pleura, pancreas and prostate. The patient was then started on azathioprine and a steroid wean commenced. Serum IgG4 levels normalized following treatment and a repeat ERCP demonstrated resolution of biliary strictures and the stent was therefore removed.

## Discussion

IgG4-related disease (IgG4-RD) is a multisystem disorder of auto-immune aetiology. It can affect a number of different organ systems and may mimic infection, inflammatory disease or malignancy.^1^ It is characterized histologically by IgG4-positive plasma cell infiltration, obliterative phlebitis and fibrosis.^2^

It is predominantly a disease of elderly males, with a male-to-female ratio of 3:1; however, the disease can also affect children.^3^ The most common manifestations of IgG4 are autoimmune pancreatitis and sialadenitis; however the disease can affect a number of different organs including the kidneys, lungs, liver, thyroid and prostate.^1^

The pathophysiological mechanism underlying IgG4 disease has not been elucidated; however, it is thought that IgG4 is not the driver of the disease, as IgG4 is unable to activate the complement cascade.^4^ Autoimmune pancreatitis was originally linked to IgG4 by Hamano et al (2001), who demonstrated higher serum concentrations of IgG4 in 20 patients with autoimmune pancreatitis, while Kamisawa et al (2003) demonstrated IgG4-positive plasma cell infiltrates on immunohistochemistry in eight patients with histologically confirmed autoimmune pancreatitis.^5,6^

Elevated serum IgG4 levels are neither sensitive nor specific for the diagnosis of IgG4-RD.^7^ Despite this, serology plays a large role in the diagnosis of this condition. Tissue immunohistochemistry remains the gold standard for the diagnosis.^1^ The diagnostic criterion for IgG4-RD focuses on the triumvirate of organ-specific clinical findings, serum IgG4 level >135 mg dl^−1^ and histological findings of IgG4-positive plasma cell infiltration.^8^

The archetypal disease process in IgG4-RD is autoimmune pancreatitis, which can be diffuse or focal. The more common, diffuse pattern of autoimmune pancreatitis is classically demonstrated on imaging as a oedematous pancreas, which can be described as sausage-shaped.^9^ The pancreas is uniformly enlarged and there is diffuse loss of the lobulated margin of the pancreas, resulting in a smooth featureless appearance.^10^ Focal autoimmune pancreas is less common but can pose a significant diagnostic dilemma. Focal autoimmune pancreatitis can present as a mass that may be difficult to differentiate from pancreatic malignancy and care must be taken to avoid unnecessary surgery.^11^ There are a number of important imaging findings which point to a diagnosis of autoimmune pancreatitis rather than pancreatic malignancy. A low attenuation or low intensity capsule-like rim evident on CT and MRI, respectively, has been demonstrated to be a reliable sign, while other findings include a smooth tapering of the upstream pancreatic duct, the so-called “icicle sign”.^12,13^ On the other hand, imaging appearances, which favour a diagnosis of pancreatic malignancy, include an abrupt cut-off in the main pancreatic duct and heterogeneous enhancement within the mass on CT.^10,12^ The presence of a peritumoural cyst has been recently demonstrated to be a specific finding for pancreatic malignancy.^14^

The lung manifestations of IgG4-RD are characterized by inflammatory lung pseudotumour, central airway disease, interstitial pneumonia and pleuritis.^15^ The diagnostic criteria for IgG4-related pulmonary disease make specific reference to pleural thickening,^8^ which was the initial finding in our patient’s case.

Prostatitis is also a recognized manifestation of IgG4-RD, with IgG4 plasma cell infiltration on immunohistochemisty, and has been shown to demonstrate increased FDG uptake on PET-CT.^16^ Due to the increased risk of prostate malignancy in elderly males, prostatic uptake should be followed-up either with repeat imaging or PSA monitoring.

PET-CT has been demonstrated to be effective in the diagnosis, evaluation of the extent of disease and evaluation of treatment response in patients with IgG4 disease.^17–19^ In 21 patients from the French IgG4 registry, all patients demonstrated increased uptake on FDG PET-CT in typical organ locations, while there was also a good correlation between PET-CT findings and treatment response.^17^ In a prospective study of 35 patients, increased FDG uptake was seen in all patients pre-treatment, and there was multisite involvement in 97% of patients.^18^ Following initial steroid therapy in the same cohort, repeat PET-CT demonstrated complete remission in 72% of patients. Although there is a good response to steroid therapy in most patients, there are a proportion of patients with poor responses and a proportion who relapse and require treatment escalation.^3^ Therefore, PET-CT can play an important role in the identification of these patients and allow for the optimization of treatment.

The treatment of IgG4-RD is oral steroids in the acute setting as standard, with the use of steroid sparing agents, such as azathioprine and methotrexate, in the long term.^1^ Although, the long-term prognosis of IgG4-RD is unknown, especially with regard to newly recognized manifestations of the disease, in particular those of the lung.^15^

In the present case, our patient was due to undergo surgical excision of the head of pancreas for a presumed malignancy. A PET-CT performed prior to this demonstrated increased uptake throughout the pleura, pancreas and prostate that led to the diagnosis of IgG4-related disease and the patient responded promptly to steroid therapy. The UGI team have become familiar with IG4-RD in the form of auto-immune pancreatitis; however, our respiratory and urology teams have less experience in these rarer sites of involvement. The radiologist has a role to play in guiding teams towards this multisystem diagnosis.

The recognition of IgG4-RD as a disease process is in its infancy. An increased awareness of this entity can lead to an earlier diagnosis for patients, thereby leading to improved outcomes. It is eminently treatable with a good response to steroids, and so early diagnosis can lead to early effective treatment and the avoidance of unnecessary interventions. Therefore, an awareness of IgG4-related disease among radiologists is essential.

## Learning points

1. The pancreas, biliary ducts and salivary glands are the most commonly affected organs in IgG4-related disease.
2. The disease can also affect the orbit, thyroid, kidney, retroperitoneum, lungs, aorta, prostate, musculoskeletal system and the skin.
3. The characteristic image findings of pancreatic IgG4-related disease is an oedematous pancreas with a halo of low density or low signal intensity. This may be associated with pancreatic or biliary dilatation and strictures.
4. There is an evolving role for PET-CT in the diagnosis, staging and evaluation of treatment response in patients with IgG4-related disease.
5. IgG4-related disease is an important differential in patients with multisystem disease. As a highly treatable condition, awareness of IgG4-RD is essential to ensure effective treatment can be initiated promptly.

## Informed consent statement

Written informed consent was obtained from the patient(s) for publication of this case report, including accompanying images.
